# Behavioral and neural measures of confidence using a novel auditory pitch identification task

**DOI:** 10.1371/journal.pone.0299784

**Published:** 2024-07-01

**Authors:** Tamara Tang, Jason Samaha, Megan A. K. Peters

**Affiliations:** 1 Department of Bioengineering, University of California, Riverside, Riverside, CA, United States of America; 2 Department of Psychology, University of California, Santa Cruz, Santa Cruz, CA, United States of America; 3 Department of Cognitive Sciences, University of California, Irvine, Irvine, CA, United States of America; 4 Program in Brain, Mind, & Consciousness, Canadian Institute for Advanced Research, Toronto, Canada; University of Miami, UNITED STATES

## Abstract

Observers can discriminate between correct versus incorrect perceptual decisions with feelings of confidence. The centro-parietal positivity build-up rate (CPP slope) has been suggested as a likely neural signature of accumulated evidence, which may guide both perceptual performance and confidence. However, CPP slope also covaries with reaction time, which also covaries with confidence in previous studies, and performance and confidence typically covary; thus, CPP slope may index signatures of perceptual performance rather than confidence per se. Moreover, perceptual metacognition—including neural correlates—has largely been studied in vision, with few exceptions. Thus, we lack understanding of domain-general neural signatures of perceptual metacognition outside vision. Here we designed a novel auditory pitch identification task and collected behavior with simultaneous 32-channel EEG in healthy adults. Participants saw two tone labels which varied in tonal distance on each trial (e.g., C vs D, C vs F), then heard a single auditory tone; they identified which label was correct and rated confidence. We found that pitch identification confidence varied with tonal distance, but performance, metacognitive sensitivity (trial-by-trial covariation of confidence with accuracy), and reaction time did not. Interestingly, however, while CPP slope covaried with performance and reaction time, it did not significantly covary with confidence. We interpret these results to mean that CPP slope is likely a signature of first-order perceptual processing and not confidence-specific signals or computations in auditory tasks. Our novel pitch identification task offers a valuable method to examine the neural correlates of auditory and domain-general perceptual confidence.

## Introduction

Metacognition is the ability to reflect on and monitor one’s own cognitive processes, including perception, memory, and decision making [1–3]—in other words, the degree to which observers’ confidence ratings discriminate between their own correct and incorrect stimulus classifications. Although the neural and computational correlates of this ability have been a topic of ongoing investigation over the past several decades [1, 4], there is still much we do not know about how metacognitive computations take place in the brain.

Noninvasive neuroimaging is a common approach to answering some of these questions in humans. In particular, studies using electroencephalography (EEG) and functional magnetic resonance imaging (fMRI) have been used to identify where and when metacognitive computations may take place. Here, we focus on the centro-parietal positivity (CPP), an EEG potential occurring approximately 300 ms post-stimulus which “ramps up” to the point at which a decision is reported. It has previously been suggested that CPP build-up rate—i.e., the speed of this ramping process—reflects the accumulation of evidence in decision-making processes, and thus can be connected to confidence within the constraints of a visual and even some auditory tasks [5–9]. The CPP build-up rate has also been suggested as a correlate of confidence judgments (albeit when not experimentally isolated from accuracy; see next paragraph) [10–12], and has been put forth as a domain-general signal unaffected by stimulus or sensory modality, unlike the P300 and P3b [6, 13]. However, some argue that the CPP and P300 are one and the same [14], so this remains an ongoing question. These studies, and the demonstrated link between CPP build-up rate and reaction time [6], provide promising evidence in line with invasive studies in nonhuman primates [15] suggesting that evidence accumulation processes in the parietal cortex may be involved in both choice and confidence computations.

However, these studies still leave open questions. First, it is known that confidence and performance typically covary, so it is unclear as to whether these previous findings reflect confidence-specific signals or signals that index overall perceptual or cognitive processing capacity [16–18]. Confidence and reaction time typically inversely covary [19, 20], and CPP build-up rate has also been found to be related to reaction time [9, 21]. Additionally, evidence has been found to continue accumulation past decision boundaries, affecting confidence judgements over time [22, 23]. Therefore, it is unknown whether EEG-based evidence accumulation signals predict confidence per se (independent of performance-driven confidence changes) versus other factors such as confidence predictions, or confidence judgements informed by past experience [24], or urgency, an evidence-independent signal that ramps up over time [13, 25]. Only one study to date has found evidence that the CPP slope predicts confidence while controlling for both reaction time and task performance [26], however this study used visual stimuli. These issues leave open the possibility that CPP build-up rate may not reflect confidence-specific signals in the auditory domain.

Confidence-based decision making/processes is hard to quantify to begin with because we are unsure whether confidence judgements are made based on the available information at the time or whether it’s indexing prior experience [24], or even the degree to which Type II confidence judgements in decisions may actually affect Type 1 decisions [27].

There is also a need to pursue the above mentioned studies outside of the visual modality, to determine domain-general signatures of confidence. Much of our knowledge of the neural and computational bases of metacognition comes from studies within vision, with a few studies venturing into auditory [28], motor [29], or multisensory [30] confidence. A great many other studies have focused on memory confidence (e.g., [31]). Most of these studies follow a similar paradigm, in which a stimulus is shown and then either immediately discriminated (plus confidence judgment) or remembered later (plus confidence judgment). Within the cognitive domain, metacognitive capacity can similarly be evaluated by asking a subject to perform a general domain knowledge task (e.g., “What is the capital of Nigeria?”) and then rate confidence [23, 28, 32]. However, to our knowledge, metacognitive judgments and their neural correlates have not been investigated in the pitch *identification*(rather than discrimination) domain. Examining pitch identification metacognition would therefore represent a novel contribution to our knowledge base about how confidence judgments in general are constructed.

In fact, pitch processing, and metacognitive evaluations of it, present a truly unique opportunity to study metacognitive evaluation given the uniqueness of the computations and neural correlates involved. For example, participants possessing absolute pitch display different ERP signatures during auditory processing than do non-absolute pitch participants [33]; this difference suggests that pitch identification may rely on neural mechanisms distinct from those involved in pitch *discrimination* capacity. This interpretation is supported by further neuroimaging studies, including observations that individuals with perfect pitch display preferential activity in the posterior dorsolateral complex, exaggerated left-lateralization of auditory processing, and absence of working memory mechanisms during pitch internal naming [34, 35]. Even on a behavioral level, previous studies have noted those with absolute pitch having difficulty making relative judgements even outside of a controlled auditory processing task environment [36–39].

Therefore, the aims of this study are twofold. First, we designed a novel pitch-identification task, as metacognitive behavior has not yet been explored in such a task. Second, we used EEG recordings to investigate how CPP build-up rates may relate to metacognition versus behavioral performance capacity in this task.

## Materials and methods

### Experimental design

#### Participants

37 healthy adult human participants gave written informed consent to participate in this experiment. The recruitment period spanned from January 15, 2020 to February 24, 2020. Ten subjects were excluded due to excessive movement leading to EEG artifacts (>70% trial loss), and an additional ten were excluded due to failure to perform the task above chance in any condition or to follow task instructions (e.g., reporting “confidence = 4” for all trials), leaving a final sample size of 17 subjects (11 females, mean age ± standard deviation 22.29 ± 5.63). Target sample size was chosen to be consistent with similar studies in the literature. All study procedures were approved by the Institutional Review Board at the University of California, Riverside.

#### Stimuli & equipment

Stimuli consisted of 24 pure sine wave tones at frequencies ranging from 261 to 988 Hz, corresponding to Western music tones and semitones between C4, or ‘middle C’, and B5. All stimuli were created and presented through custom scripts in PsychToolbox [40, 41] presented via Matlab (The Mathworks, Inc., Natick, MA). Auditory stimuli were played via speakers placed on the desktop at a comfortable listening volume.

A Dell computer running Windows 10 Pro 64k and MATLAB R2019a controlled the experiment. Participants sat approximately 45–50-in. from the screen with their chins in a chinrest to minimize movement. Instructions and response prompts were presented on an NEC MultiSync FE2111SB CRT monitor (75 Hz refresh rate, 1280x1024 resolution). Mediating the connection between the monitor, the computer, and the EEG system was a DATAPixx processor (VPixx Technologies, Inc., Saint-Bruno, QC, Canada) which facilitates monitor-refresh-locked stimulus presentations for accurate timing of stimuli and response triggers.

#### Behavioral task

**Fig 1:**
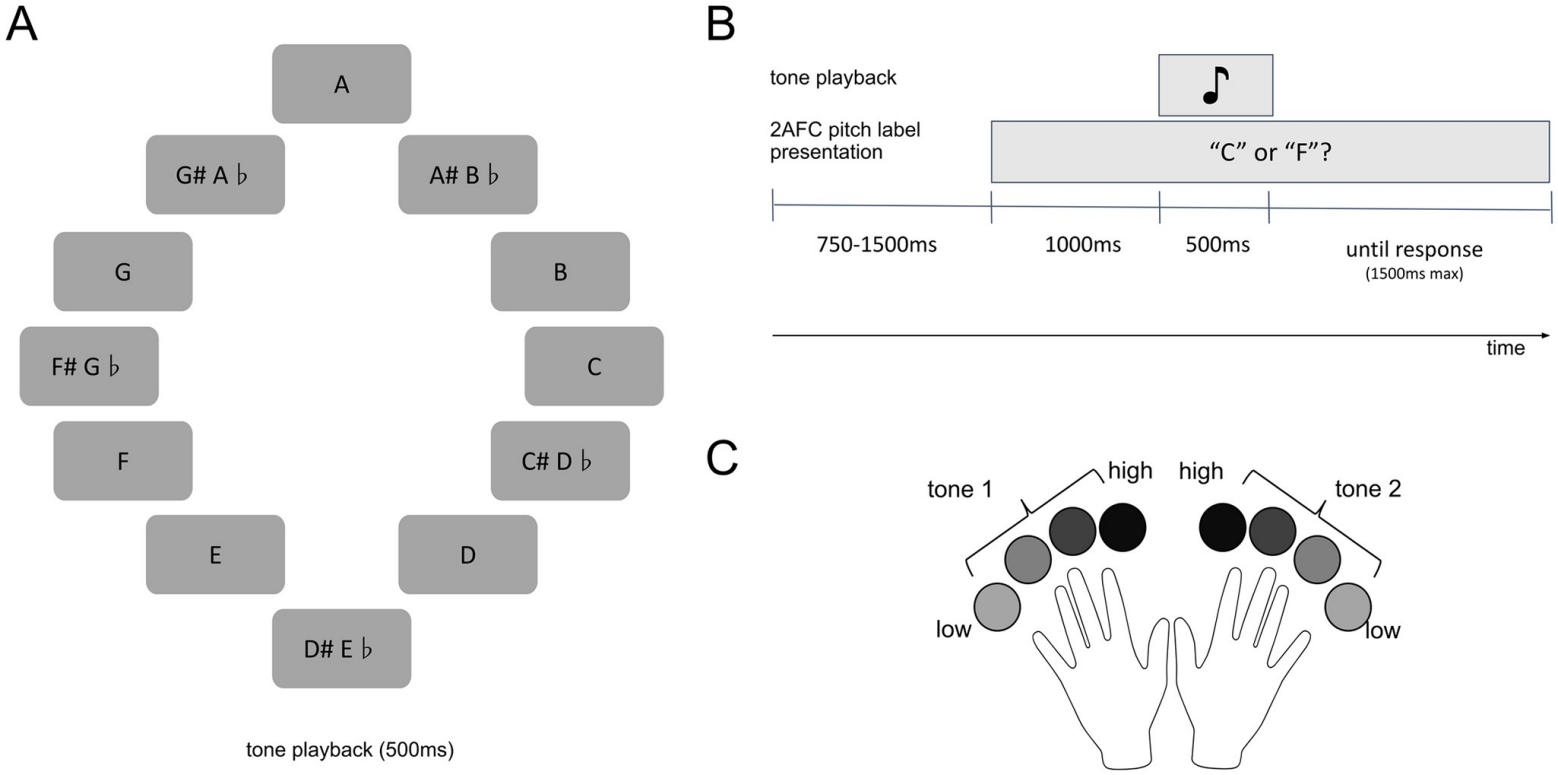
Behavioral task structure. **(A)** Possible pitches from which two tones were pseudorandomly drawn and presented during the 2AFC pitch label presentation. **(B)** Example trial structure showing one possible pitch pairing (see Table 1 for description of tonal distance pairings). **(C)** Responses for choice and confidence were made with a single simultaneous button press.

The behavioral task was a pitch-identification 2-alternative forced-choice (2AFC). Subjects were visually presented with two possible tone labels (e.g., “C or F?”; Fig 1A), and then heard a single pure tone played through headphones; participants had to select which label matched the tone that they heard and rate their confidence on a scale of 1–4. Visual presentation of the tone labels was shown for a duration of 1000 ms concurrent with a fixation dot (to minimize eye movements), followed by tone playback for 500ms followed by a 1.5s response window; tone labels remained on the screen throughout tone playback and the response window (Fig 1B). Subjects entered their tone choice and confidence rating simultaneously with a single button press on each trial (Fig 1C).

**Table 1:** Definition of *tonal distance bins*, with examples and percentage of all trials that each bin comprises.

Bin	1	2	3	4	5	6
**Semitonal distance**	1	2	3	4	5	6
**Examples**	A & A# G# & A	A & B G & A	A & C F# & A	A & C# F & A	A & D E & A	A & D#
**% of total trials**	18	18	18	18	18	10

Trials were presented in pseudorandom order, such that on every trial a random pair of tone labels was drawn, one (the target) corresponding to the pure sine tone that was subsequently played and the other (the distractor) drawn from the remaining 23 sine tones. Participants completed approximately 1300 trials (10 blocks of $\tilde{1}30$ trials each, distributed evenly across the 24 semitones) in a single session which lasted about 2 hours. Prior to the main task, subjects undertook sufficient practice blocks (typically about 5) to learn the task (subjects moved from practice to main task when they verbally reported that they understood the task).

#### Questionnaires

Musical aptitude was determined through self-assessment as well as objective quantification of musical sophistication via the Goldsmith Musical Sophistication Index [42] presented prior to the behavioral task. Subjects self-reported levels of musical training on a scale of: no musical training or experience, some musical training or experience (amateur to professional), or musical training. Subjects also self-reported whether they possessed absolute pitch, as well as number of languages spoken, age at which they began formal musical training, number of years of musical training, average musical training hours per week, and their primary instrument. All subjects completed these questionnaires prior to engaging in the behavioral task. Subjects who claimed to possess absolute pitch were then given an additional diagnostic test (http://www.musicianbrain.com/aptest/) and were required to surpass 90% within a range of one semitone to qualify as having true absolute pitch. Due to data loss (see Participants), however, we did not have enough complete datasets to facilitate individual differences analyses with these data and so musical training results are not reported in this manuscript.

#### Imaging

While subjects performed the behavioral task, 32-channel EEG data (Ag/AgCl) were recorded using a BrainVision ActiCap system (Brain Products GmbH, Munich, Germany) via the BrainVision Recorder software (Version 1.21.0303, Brain Products GmbH, Munich, Germany) at 500 Hz sampling frequency. All electrodes were mounted in an elastic cap according to the international 10/20 system [43], and were grounded with a forehead ground and online referenced to Cz. Impedances were kept below 20 kΩ on average, and an antialiasing low-pass filter of 245 Hz was applied via an ActiCHamp amplifier (Brain Products GmbH, Munich, Germany). Conductive gel was applied to maintain the contact between the electrodes and the scalp. Event and response triggers were synchronized with the recorded EEG signal via TTL signals sent through the VPixx DATAPixx system.

### Analysis

#### Behavioral analysis

We calculated a number of behavioral metrics, including measures of task performance, confidence, and metacognitive sensitivity. Each metric (described below) was computed for each subject within *tonal distance bins*, defined as the absolute distance between two semitones (target and distractor) within a single 12-semitone octave. The bins were therefore defined as:

**Type 1 performance.** First, we quantified the type 1 performance of each observer in each tonal distance bin with two performance measures: percent correct responses, and the signal detection theoretic metric d’ [44, 45]. d’ is defined as d’=z(HR)-z(FAR), where z(⋅) refers to the inverse normal transform, and HR and FAR are the hit and false alarm rates, respectively.

**Confidence.** Participants rated confidence on a scale of 1–4 (see Behavioral task), but it is likely that different participants defined each confidence rating differently. Therefore, to standardize ratings across observers, we computed the within-subject z-score of confidence, z(confidence), with $z\left(x\right)=\frac{x-mean(x)}{std(x)}$. We computed z(confidence) in each tonal distance bin for each observer. (Note the difference here between z-score and the inverse normal transform in the previous section.) Whenever we refer to ‘confidence’ throughout the Results and Discussion sections, it is thus this z(confidence) measure.

**Metacognitive sensitivity.** We are also interested in quantifying metacognitive sensitivity, i.e. the degree to which confidence can covary with accuracy on a trial-by-trial basis. We used three metrics to quantify metacognitive sensitivity: meta-d’ and M-ratio, which are type 2 performance measures standardized to the same scale as type 1 d’ [2, 46, 47], and area under the type 2 receiver operating characteristic curve (confAUC) [1, 48]. These are standard metrics used in studies of perceptual metacognition [1]. Meta-d’ and M-ratio were computed using the MLE meta-d’ toolbox [2] for each tonal distance bin. ConfAUC was calculated for each tonal distance bin according to standard approaches [1].

**Reaction time.** Reaction time was defined as the difference between tone onset and the button press response for the 2AFC decision (i.e. tone 1 or tone 2?). As done for confidence, to standardize across subjects and control for individual differences in overall reaction time, we also z-scored reaction time within each subject across all trials by first log transforming the raw reaction time then calculating z(log(reaction time)), where again z(x)=x-median(x)std(x). (Note here that this is not precisely a z-score as it uses median rather than mean; however, it still functions as a standardization of reaction times across participants.) Whenever we refer to ‘reaction time’ throughout the Results and Discussion sections, it is thus this z(log(reaction time)) measure.

**Statistical analysis.** Relationships between tonal distance bin and each of the above behavioral metrics were quantified via one-way repeated measures ANOVAs with the single factor of tonal distance bin. Thus, we asked the following questions:

1.1 Does percent correct choices change with tonal distance bin?
1.2 Does d’ change with tonal distance bin?
1.3 Does M-ratio change with tonal distance bin?
1.4 Does confidence change with tonal distance bin?
1.5 Does reaction time change with tonal distance bin?

We also explored relationships between confidence and behavior on a trial-by-trial basis using the area under the ROC curve (AUC), Spearman, and partial Spearman correlations (bin is an ordinal variable, so Pearson correlations are inappropriate).

2.1 Does confidence covary with performance (trial-wise correct/incorrect)?
2.2 Does confidence covary with reaction time?
2.3 Does confidence covary with performance while controlling for tonal distance bin?

For all statistical analyses, outliers greater than 3 standard deviations from the group mean were ignored. Statistical analysis on all behavioral metrics were performed using MATLAB 2019b.

#### EEG data processing

*Pre-processing.* EEG data were preprocessed and ERP analysis was performed through MATLAB 2019b and the EEGLAB toolbox. Data were high-pass filtered at 0.1 Hz using a basic FIR filter and re-referenced to the average of the left and right mastoids. For ERP analysis, data were epoched at tone onset with a window of -1000 ms to 2000 ms and baselined using a 500 ms pre-stimulus window. Eye blinks and muscle artifacts were removed through independent component analysis (ICA), and additional visual inspection was performed to filter out trials contaminated by excessively noisy data, residual eye movements, extraneous movements, and blinks. Some trials included in the behavioral-only analysis were removed for the ERP analysis to isolate clean trials; we made the choice to keep these trials in the behavioral-only analysis in order to maximize trial counts. On average, approximately 30% of trials were removed from the EEG analyses due to noisy EEG data.

*EEG statistical analysis.* ERP analysis was conducted using the MATLAB 2019b EEGLAB toolbox. We focused on the CPP region (i.e. Pz, P3, P4), for a reflection of the accumulation of perceptual evidence towards decision making [5, 49]. ERPs were computed in both stimulus-locked and response-locked fashion—the former for quality check and to examine differences in ERP magnitude across tonal distance bin, and the latter to also facilitate calculation of the CPP build-up rate, a proxy for evidence accumulation [6, 8]. Stimulus-locked ERP analysis was defined within a 1000 ms time window of -500 to 500 ms, centered with respect to stimulus onset. Response-locked ERP analysis was defined within a 1000 ms time window of -500 to 500 ms, centered with respect to trial-by-trial response onset. We also repeated the ERP analyses for the Pz electrode alone due to its noted correlation with type 2 processing [6], with similar results.

We also analyzed CPP build-up rates on a trial-by-trial basis. CPP build-up rate is defined as the slope of a straight line fitted to the response-locked waveform on each trial, with a time window defined individually for each subject as 250 ms pre-response [6, 9, 21, 49, 50]. Trial-by-trial CPP build-up rate was first standardized by z-scoring all CPP build-up rates within each subject across all trials, such that if one subject had overall faster CPP build-up rates this would not contribute to increased between-subjects variance on a condition-specific basis. For the main analysis, we grouped CPP build-up rates by each tonal distance bin across all trials in that bin and performed an ANOVA to determine whether CPP slope changed as an effect of tonal distance bin. We also looked for relationships between CPP slope while binned by % correct or confidence rather than tonal distance bin via t-test and one-way repeated measures ANOVAs.

Thus, following on the previous questions sets, we ask:

1.6 Does CPP build-up rate change with tonal distance bin?

Finally, we looked at the effect of CPP slope on a trial-by-trial basis. Spearman correlations across all trials within each subject were calculated to determine a significant effect of CPP slope with behavioral measures, such as performance, z(confidence) and log(reaction time). To further confirm a significant effect on confidence and tonal distance bin, we performed additional partial correlations while controlling for performance, tonal distance bin, or both.

2.4 Does CPP slope covary with performance (trial-wise correct/incorrect)?
2.5 Does CPP slope covary with confidence?
2.6 Does CPP slope covary with reaction time?
2.7 Does CPP slope covary with confidence while controlling for performance?
2.8 Does CPP slope covary with performance while controlling for tonal distance bin?
2.9 Does CPP slope covary with confidence while controlling for both tonal distance bin?
2.10 Does CPP slope covary with confidence while controlling for both tonal distance bin *and* performance?

As a quality check on these analyses, we then grouped CPP build-up rates into “slow”, “medium”, and “fast” reaction times by binning trials’ normalized reaction times into bins defined as below the 33rd percentile, between the 33rd and 66th percentile, and above the 66th percentile within each participant; we then visualized these results in a bar graph.

## Results

### Behavioral results

#### Tonal distance bin analysis

**Fig 2:**
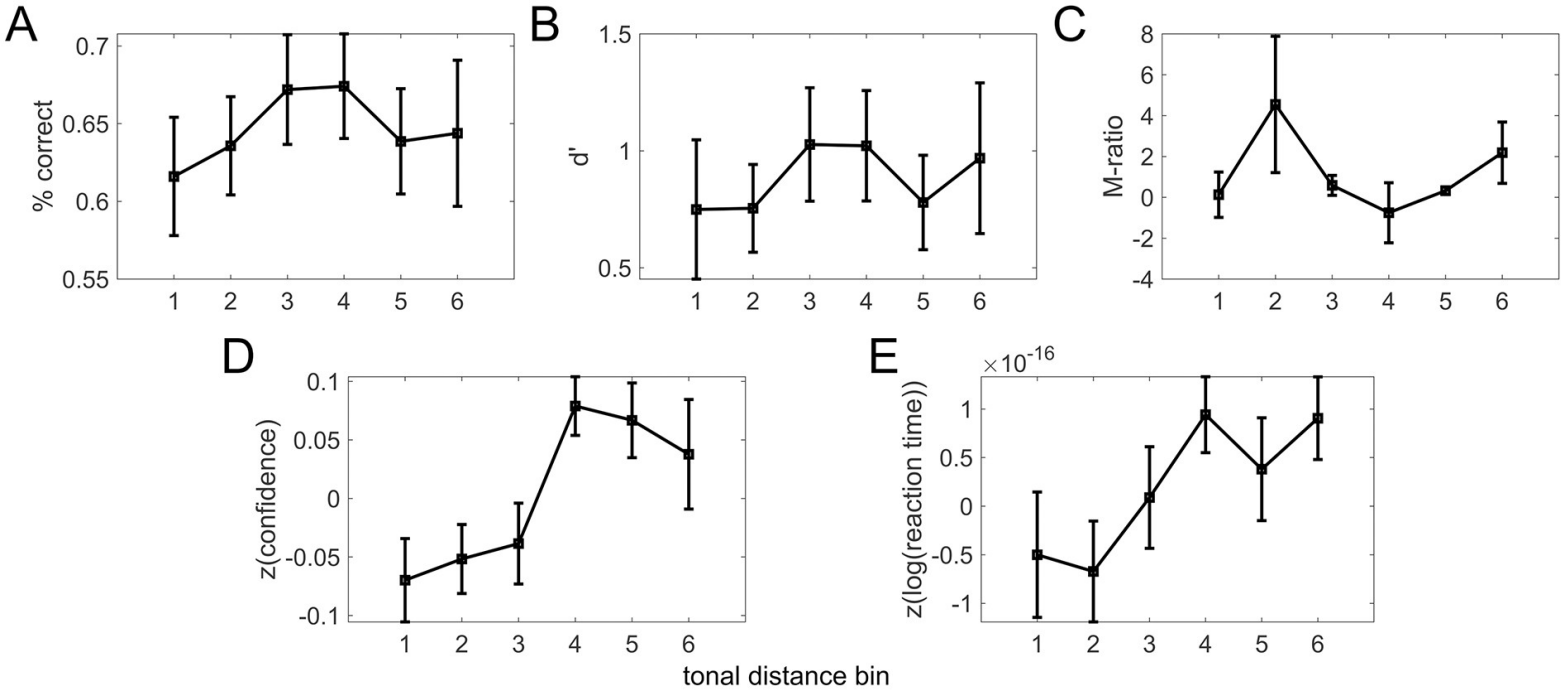
Relationships between tonal distance bin (TDbin) and behavioral measures. (A, B, C, D, E) for % correct, d’, M-ratio, z(confidence), z(log(reaction time)), respectively. We performed one-way repeated measures ANOVAs to quantify these effects. See Table 2 for statistical results.

From our binned analysis using ANOVAs, we observed no significant main effects of tonal distance bin on measures of type 1 performance, d’ and percent correct (Fig 2A and 2B, Table 2). This means that the tonal distance bin did not affect type 1 task performance. This may be because the participant is visually presented with 2 tones but hears only a single auditory tone, making it less likely that they could achieve differentially above-chance performance on the task as a function of the visual stimuli changing unless the participant possessed either absolute pitch or high musical aptitude.

**Table 2:** ANOVA results for behavioral measures by tonal distance bin.

Question #	… vs. TD bin	F-value	Num df	Den df	p-value
1.1	% correct	1.57	5	80	0.178
1.2	*d*′	0.8	5	75	0.552
1.3	M-ratio	1.88	5	75	0.109
1.4	*z*(confidence)	2.81	5	80	0.022*
1.5	*z*(*log*(reaction time)	1.74	5	80	0.137

* *p* < 0.05

In contrast to the performance measures, we observed a significant main effect of tonal distance bin on confidence (Fig 2D, Table 2). This implies that subjective confidence ratings increase with tonal distance bin. This is perhaps surprising given that performance and confidence typically covary (e.g., [9, 28]); however, we did see this typical relationship in the trial-by-trial analyses (see Behavioral results). Regarding metacognitive sensitivity, we observed no apparent relationship between tonal distance bin and M-ratio (Fig 2C, Table 2). However, this is not surprising since M-ratio is a measure of metacognitive sensitivity that purposefully corrects for type 1 performance capacity. Although subjects were reporting feeling more confident in larger tonal distance bins, their capacity to track their trial-by-trial accuracy with their reported confidence did not change with tonal distance bin. We note that the M-ratio values observed here are also quite extreme relative to what might be expected based on theoretical grounds and previous studies (i.e., M-ratio values around 1). While it is perhaps surprising to observe M-ratios so extreme, we believe these estimates are due to the small number of trials in our dataset and the idiosyncrasies of this particular task; that is, we do not believe that M-ratios that appear much larger than 1 should be taken to mean that subjects had “super metacognition” in those TD bins, but rather that estimating M-ratio is sensitive to small trial counts especially in this task. We also note that the M-ratio magnitudes observed here are highly variable due to some very small or even negative values of d’, which is in the denominator of the measure leading to inflated (or highly negative) M-ratio values.

Finally, we did not observe a significant relationship between tonal distance bin and reaction time (Fig 2E, Table 2). In concert with the observation that confidence did in fact vary with tonal distance bin, this may seem somewhat surprising, as performance, confidence, and reaction time typically do covary [20]. We note that these relationships are more explored later in the trial-by-trial analyses in Behavioral results.

In sum, we observed that task performance does not vary with tonal distance bin, but confidence does. We also observed that metacognitive sensitivity and reaction time do not. We were next interested to find out how these measures covaried on a trial-by-trial basis.

#### Trial-by-trial analysis

**Fig 3:**
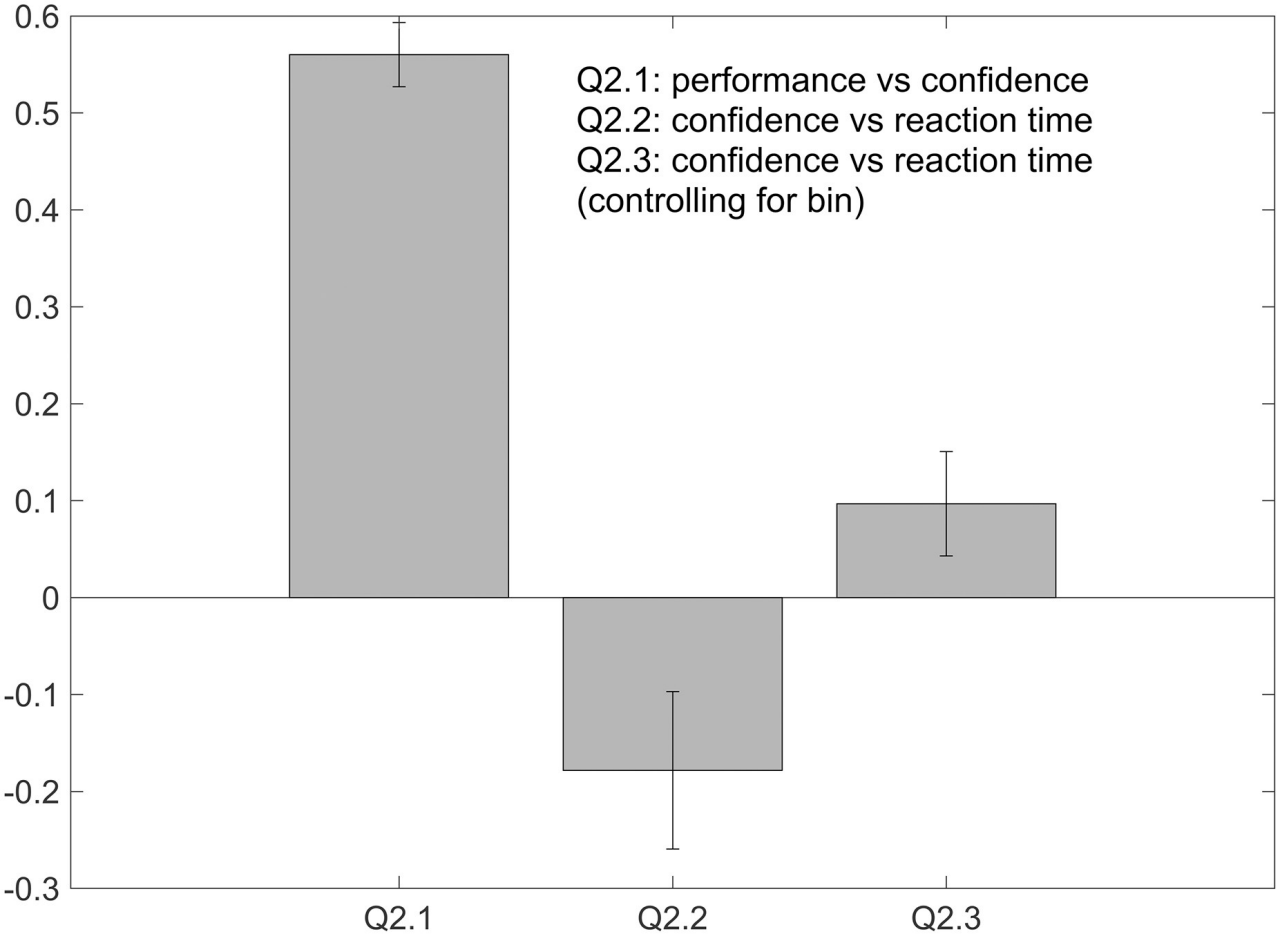
Average metrics for normalized confidence versus behavioral measures, answering Questions 2.1—2.3. Each measure is different (refer to main text), capturing the quantitative relationship between a unique pair of behavioral measures, so the y-axis is unlabeled: Q2.1 shows area under the ROC curve, Q2.2 shows Spearman R, and Q2.3 shows partial Spearman R. See Table 3 for details and statistics.

We found a significant effect between confidence and type 1 performance (correct/incorrect; Q2.1) (Fig 3, left bar; Table 3). This further supports previous findings that confidence and performance covary in an auditory domain [9, 28]. We also found confidence to be inversely correlated with reaction time (Q2.2; Fig 3, middle bar; Table 3). This would make sense that reaction time decreases with increasing confidence ratings. This relationship carried through even while controlling for the effect of tonal distance bin (Q2.3; Fig 3, right bar; Table 3), which is interesting when considering we were unable to find an effect of reaction time when binned by tonal distance. We might interpret this to mean that reaction time by itself is not related to tonal distance, but is connected more to performance and confidence.

**Table 3:** Average Spearman correlations and t-test results for trial-by-trial measures.

Question #	Pair of measures	Analysis	Average metric	t(df = 16)	p-value
2.1	*z*(confidence) vs. correct/incorrect	AUC	0.560	4.318	< 0.001**
2.2	*z*(confidence) vs. *z*(*log*(reaction time)	Spearman *R*	-0.178	-5.226	< 0.001**
2.3	*z*(confidence) vs. performance (controlling for bin)	Partial Spearman *R*	0.097	4.281	< 0.001**

All t-tests against 0, except for Q2.1 (AUC) which was against 0.5.

* *p* < 0.05,

** *p* < 0.01.

### EEG results

#### ERP results

**Fig 4:**
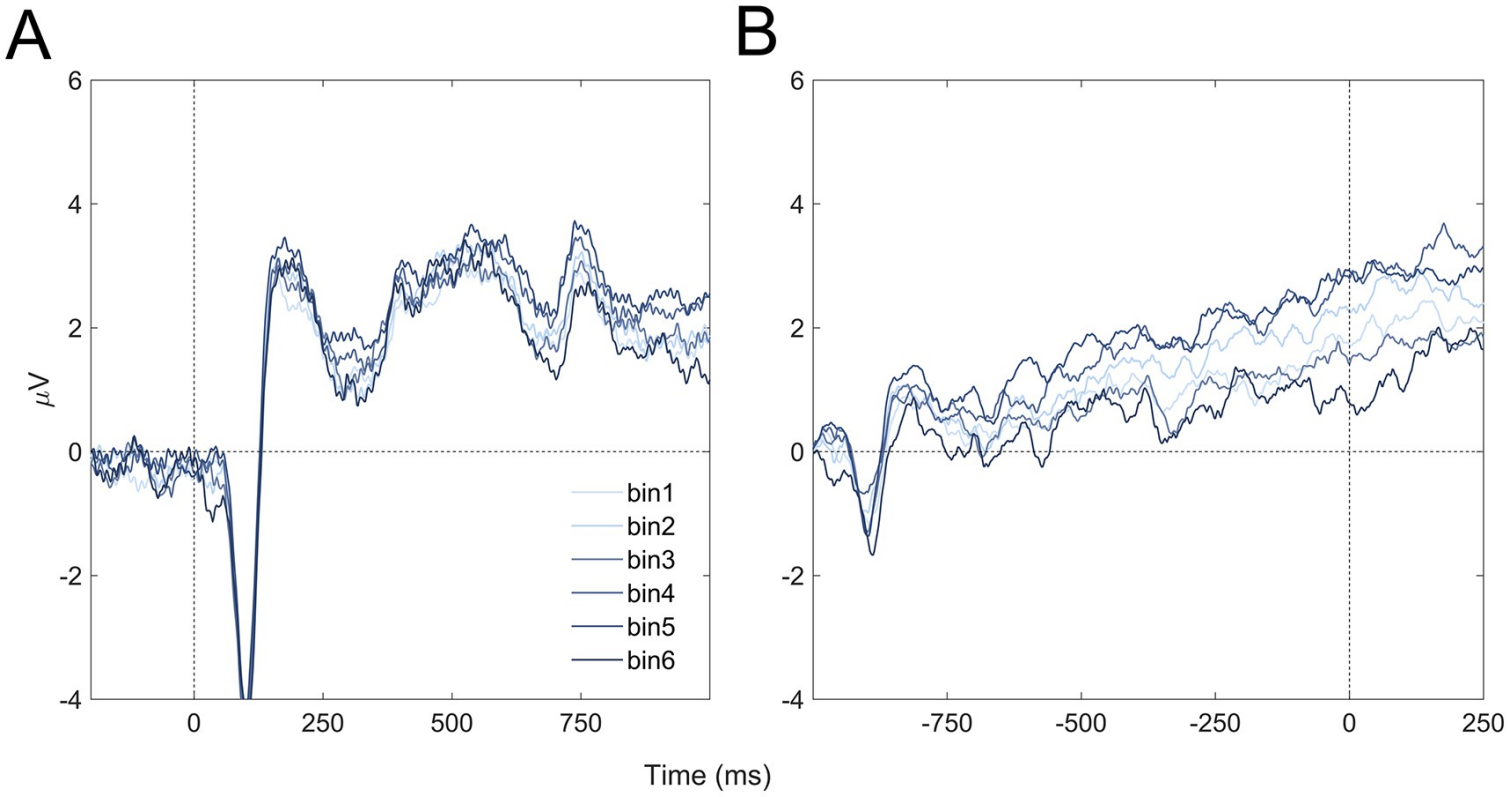
ERPs as a function of tonal distance bin. **(A)** Stimulus-locked ERP of CPP region (Pz, P3, P4). **(B)** Response-locked ERP of CPP region.

We first computed the stimulus-locked and response-locked ERPs of the CPP region (i.e. Pz, P3, and P4) to visually confirm our preprocessing pipeline (see Materials and methods for details)). In line with standard findings [51], we observed a clear N1 at approximately 100 ms and clear P2 at approximately 150 ms after tone onset in the stimulus-locked analyses (Fig 4A), and a ramping behavior towards reaction time onset in response-locked analyses (Fig 4B).

#### CPP results

**Fig 5:**
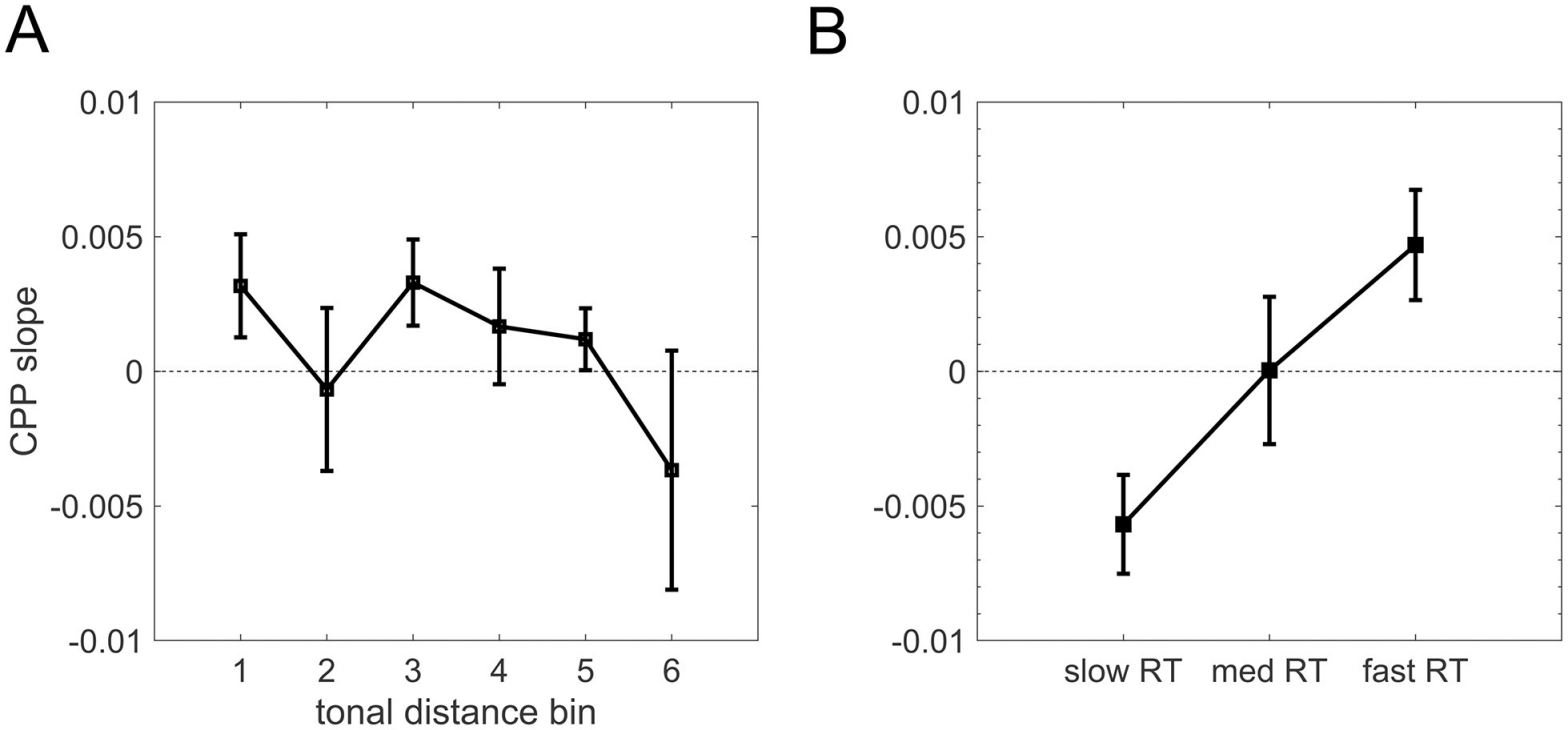
CPP slope as a function of tonal distance bin and reaction time. **(A)** Relationship between tonal distance bin and standardized CPP slope (see main text for statistics). **(B)** Normalized CPP build-up rates of slow, medium, and fast z(log(reaction time) for all participants. Normalized CPP slope showed a meaningful covariation with reaction time, with faster RTs associated with steeper CPP slopes.

*ANOVAs with CPP build-up rate for TD bin.* Our critical analysis examined CPP build-up rates in the response-locked data because it has been previously argued that the slope of the CPP reflects the build-up of evidence accumulation and is closely related to confidence in a decision-making task [6, 9, 21, 49, 50]. However, previous research did not control for task performance capacity. We therefore calculated the response-locked CPP build-up rate for all subjects (see Materials and methods) across the CPP region (electrodes Pz, P3, and P4). To confirm that this analysis correctly identified CPP build-up rate, we then binned the results into slow, medium, and fast reaction time trials (defined within-subject). Results showed that the standardized CPP build-up rate significantly covaried with reaction time in the expected way (Fig 5B; Table 3) (here we used Pearson correlations because in order to do a t-test, you must Fisher-Z transform the correlation coefficients and Spearman correlation coefficients were often 1, leading to infinite values after the Fisher-Z transformation). This validation allowed us to proceed with our main analysis of interest: examining whether CPP build-up rate related to confidence alone (or other metacognitive measures) rather than performance-driven confidence.

We first calculated an ANOVA for standardized CPP build-up rate separately for trials within each tonal distance bin and did not find any significant effect between tonal distance bin and CPP slope (F(5,80) = 1.08, p = 0.375). While standardized CPP slope sat just above zero for bins 1, 3, 4, and 5, we observe a slightly negative standardized CPP slope for bin 6, and a standardized CPP slope of about 0 for bin 2.

**Fig 6:**
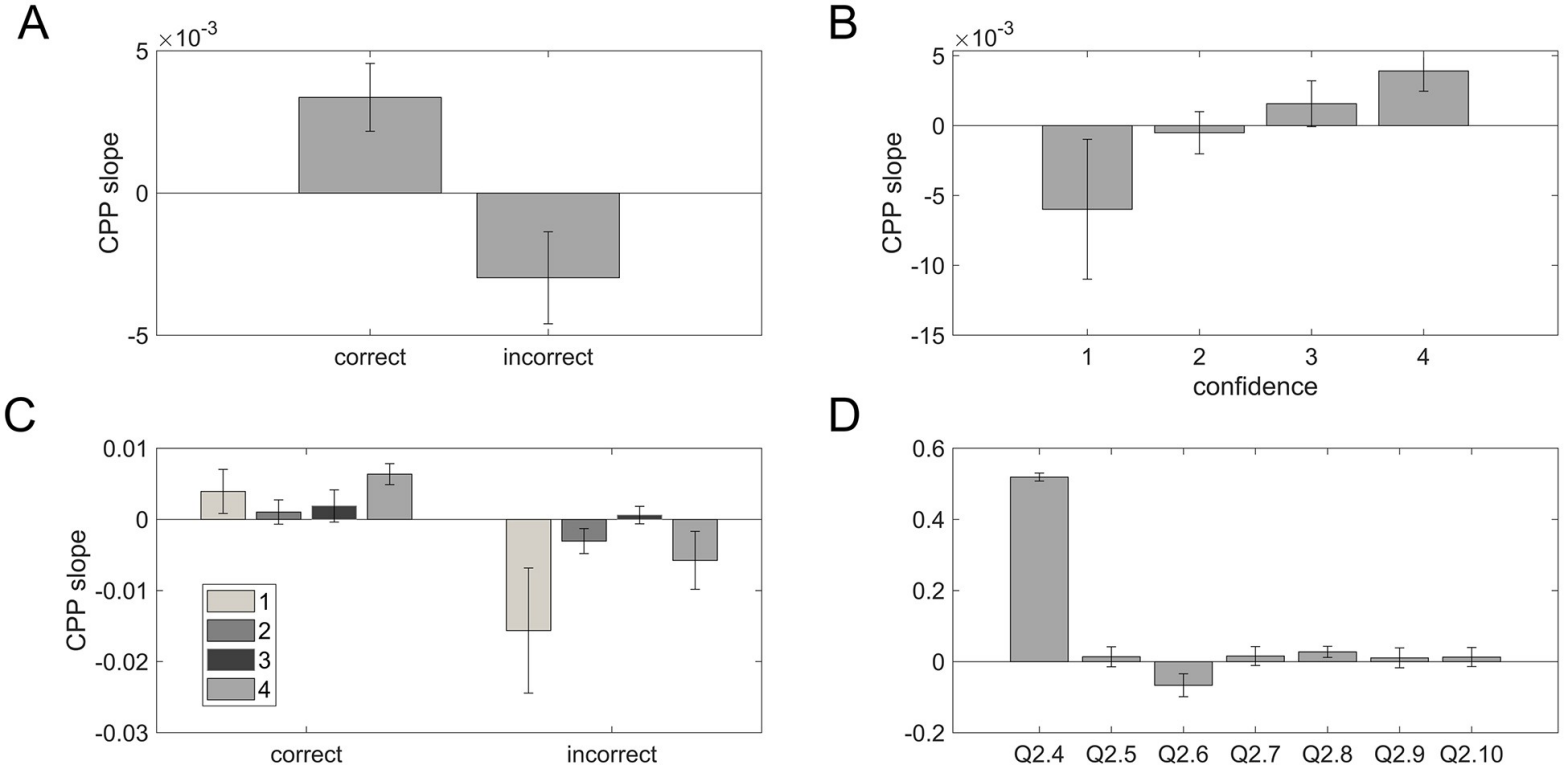
Plots answering Questions 2.4–2.10: Trial-by-trial analyses between CPP slope and behavioral measures. Each bar shows the quantitative metric for a different question or relationship among variables. **(A)** Mean CPP slope as a function of correct vs incorrect. **(B)** Mean CPP slope as a function of confidence level (1–4). **(C)** CPP slope as a function of % correct binned by confidence level. **(D)** Mean value of trial-by-trial correlations. See Table 4 for statistical outcomes.

*Trial-by-trial analysis.* We also sought to identify an effect of CPP slope when separated by various factors other than tonal distance bin. We found a positive CPP slope for correct responses, while there is a negative CPP slope for incorrect responses (t(16) = 3.6285, p = 0.0023) (Fig 6A). When separated by confidence, we see a linear ramp up of CPP slope by confidence level, with a steep negative CPP slope when confidence = 1 and a positive CPP slope when confidence = 4 (Fig 6B); however, this did not reach statistical significance (F(3,48) = 2.45, p = 0.075). Finally, it is important to parcel out the effect of correctness on confidence, so we analyzed CPP slope binned by both correct vs. incorrect and confidence; this analysis revealed again a main effect of correctness (F(1,13) = 9.136, p = 0.010) but no main effect of confidence (F(3,39) = 0.459, p = 0.713) and no interaction (F(3,39) = 2.006, p = 0.129)(Fig 6C).

We then calculated AUC, and Spearman and partial Spearman correlations across all trials within each subject to determine whether CPP build-up rate covaried with performance, confidence, or other metacognitive measures. We found a significant effect between CPP slope and type 1 performance (correct/incorrect, Q2.4) (Fig 6D, Table 4). This relationship carried through even while controlling for the effect of tonal distance bin (Q2.8). CPP build-up also has a significant inverse relationship with reaction time; as reaction time decreases, we see a ramp up in evidence accumulation (Q2.6).

**Table 4:** Average Spearman correlations and t-test results for CPP slope and trial-by-trial measures.

Question #	Pair of measures	Analysis	Average metric	t(df = 16)	p-value
2.4	CPP slope vs. correct/incorrect	AUC	0.519	2.645	0.0054**
2.5	CPP slope vs. *z*(confidence)	Spearman R	0.013739	0.7603	0.4581
2.6	CPP slope vs. *z*(*log*(reaction time)	Spearman R	-0.066412	-3.2139	<0.01**
2.7	CPP slope vs. *z*(confidence) (controlling for correct/incorrect)	Partial Spearman R	0.015722	0.9161	0.3732
2.8	CPP slope vs. correct/incorrect (controlling for bin)	Partial Spearman R	0.02739	2.7556	0.0141**
2.9	CPP slope vs. *z*(confidence) (controlling for bin)	Partial Spearman R	0.010644	0.59093	0.5628
2.10	CPP slope vs. *z*(confidence) (controlling for bin and correct/incorrect)	Partial Spearman R	0.012952	0.75502	0.4612

All t-tests against 0, except for Q2.4 (AUC) which was against 0.5.

* *p* < 0.05,

** *p* < 0.01

We did not find any relationship between CPP slope and confidence (Q2.5), nor did any effect appear when controlling for performance (Q2.7), tonal distance bin (Q2.9), or both measures at the same time (Q2.10). Recall from our behavioral analysis that we found performance and reaction time to covary with confidence, and performance covaries with CPP slope, but confidence and CPP slope do not covary with each other. Thus, we see that CPP slope appears to be related to evidence accumulation that directly contributes to performance on the task, but bears no relation to metacognitive measures in the auditory domain.

## Discussion

Here, we sought to investigate the domain-general neural signatures of metacognition using a novel 2-alternative forced choice auditory pitch identification task paired with 32-channel EEG recording. We focused on behavioral measures of performance, confidence, and metacognitive efficiency both grouped by condition and trial-by-trial, as well as neural correlates of evidence accumulation and decision-making.

We found that task performance (% correct and d’) did not change as a function of tonal distance bin (the tonal distance between target and distractor tones displayed on the screen; Fig 1). Although this may appear initially surprising, we expect that these findings are due to the nature of our task as a tone-identification task, meaning that the difficulty of the tone identification task may not depend so greatly on the visual presentation of two tone labels as would an auditory tone discrimination task (for example). We also found that M-ratio, a measure of trial-by-trial correspondence between confidence and accuracy that is corrected for task performance capacity [2, 46] did not vary with tonal distance bin, nor did reaction time. However, we found that confidence did significantly vary with tonal distance bin. On a trial-by-trial basis, we found that performance was significantly related to confidence, even after controlling for the effect of tonal distance bin; both measures were significantly related to normalized reaction time.

Crucially, we also examined the relationship between centro-parietal positivity (CPP) build-up rate (i.e., CPP slope) and behavioral measures. For this, we were inspired by the observation that CPP build-up is often related to reaction time [9, 21], but confidence and reaction time also typically inversely covary [20]. Therefore, we wanted to examine the specific relationship between confidence and CPP slope beyond its relationship with reaction time or performance. Though we did not find CPP slope to change significantly as a function of tonal distance bin, we observed that CPP slope meaningfully covaried with reaction time on a trial-by-trial basis, such that CPP slope was bigger for faster reaction times as expected. We also saw that CPP slope covaried with measures of task performance. However, there does not seem to be any significant covariation between CPP slope and confidence. We interpret this to imply that CPP slope appears to index neural signatures of evidence accumulation that directly relate to task performance capacity, but may not include information about confidence per se in this auditory task.

We believe these findings also to be important because of the paradigm-based approach for controlling performance. Although we did not explicitly manipulate our conditions so as to hold performance constant, nevertheless our results showed several conditions with invariant performance yet differing confidence judgments. Previous approaches to investigating confidence while paradigmatically controlling for performance have highlighted the importance of avoiding cases where the two measures are highly correlated across conditions (e.g., [18, 52, 53]), and methods such as post-hoc trial selection are undesirable for other reasons [54]. Take, for example, a hypothetical extreme case where in Condition A, the observer is performing at chance (50% correct), and in Condition B, the observer is performing at near-ceiling (90% correct). Then, say that one analyzes only the correct trials from both conditions. It is clear that the correct trials in Condition A are only correct because of a random guess, whereas in Condition B, the correct trials are correct because the observer actually meaningfully processed the stimulus. This is why post-hoc selection of correct-only trials is not an appropriate way to control for performance confounds in analyzing computations or their neural correlates, and why designs that show performance-matching—such as the one explored here—are better suited to isolating neural and/or computational processes associated with confidence per se.

Our findings also contribute to the broader literature regarding the behavioral signatures and neural correlates of metacognition in perception. Specifically, the vast majority of studies on perceptual metacognition have been conducted in the visual modality, and those which observe separable neural signatures of performance versus confidence in EEG recordings are few. For example, Balsdon and colleagues [55] used a visual task and EEG to reveal that unique confidence representations can be observed in superior parietal and orbitofrontal cortices, even after decisions have been committed to. Tagliabue and colleagues [49] used visual stimuli at varying levels of visual stimulus intensity to demonstrate the linkage between subjective conscious experience and neural decision-making processes.

However, only a few studies specifically examine non-visual confidence, including in the auditory [28], motor [29], or multisensory [30] domains. In tactile sensation, for example, it has been reported that the CPP tracks both subjective decision evidence and confidence [11], but the task used by those authors did not specifically control for perceptual performance. Because confidence and performance typically covary, one would ideally want to control for performance while looking for neural signatures of confidence more specifically [16–18]. Likewise, in the auditory domain, Zakrzewski and colleagues [28] used a 2-interval forced choice detection paradigm to examine ERP signatures of auditory pitch detection and confidence ratings. They reported that N1, P2, and P3 amplitudes all covaried with confidence ratings, while P3-like late positivity was higher in amplitude in lower confidence, target-absent intervals. They interpreted their results to mean that metacognitive judgments could track both sensory- and decision-related processes, but that the processes driving confidence likely depended on task. However, note that these results are consistent with the broader literature linking N1-P2 auditory evoked potentials to auditory processing at a type 1 level [51, 56], and, as above, Zakrezewski and colleagues did not control for performance differences. Thus, the correlates they revealed may have primarily indexed sensory or type 1 processing and not metacognition per se, as also suggested above. Thus, there is a need to pursue the domain-general signatures of perceptual confidence independent of those for performance, especially outside the visual modality.

There are several possible reasons why our results were more equivocal regarding the relationship between CPP slope and confidence. First, our trial counts were limited due to data cleaning procedures, resulting in up to 30% of trials being discarded for many subjects for movement or other artifacts. This lack of statistical power may have obscured a meaningful relationship between CPP slope and confidence, should they have actually been present. Due to Covid-19 shutdown of research when these data were being collected, we also were unfortunately unable to recruit enough participants possessing absolute pitch, or enough variation in musical aptitude, to reveal any meaningful relationships between these abilities and the behavioral or neural measures used here. However, we also note that despite our low trial counts and participant numbers, we were able to observe meaningful trial-by-trial relationships between CPP slope and two different measures of performance (percent correct and d’), as well as confirm standard relationships between CPP slope and reaction time; therefore, we believe these 2concerns are not primarily responsible for the lack of CPP relationship with confidence or with other measures of metacognition.

We also note that with more participants, it might also have been possible to use a different measure that might be more sensitive to the correlation between musical aptitude and task performance and/or perceptual metacognitive measures, as well as the EEG signatures of these. We were initially inspired by the observation that participants who have absolute pitch show different auditory processing ERP signatures than those who do not [33]. Later behavioral [36–39] and neuroimaging studies [34, 35] have supported the interpretation that absolute pitch processing might be categorically different from pitch discrimination processing in general [33, 35, 57]. These observations suggested to us that pitch identification might be a particularly informative approach to understanding domain-general metacognitive signatures using EEG. For the purposes of this study, we relied on the main analyses commonly done in previous studies [49, 57–59], and unfortunately were unable to collect a varied enough sample to be able to undertake this analysis. Despite these limitations, our findings demonstrate that our novel pitch-identification task does reveal interesting and potentially powerful insight into the neural signatures of performance and confidence in the auditory domain, leading to better insight about domain-general metacognitive processing. Future studies should expand on the data and analyses presented here to further contribute to knowledge about auditory metacognition.

Finally, we note that because tone playback was fixed in this task to be 500ms, followed by a response period (see Materials and methods), reaction times here may not have been as informative as had it been a true reaction time task. Nevertheless, we did observe significant trial-by-trial covariation between reaction time and confidence, reaction time and performance, and reaction time and CPP slope. Therefore, reaction time was still informative even though our stimuli were presented at fixed durations.

Together, our results present a novel auditory pitch identification task plus EEG analyses as a step towards identifying domain-general signatures of perceptual metacognition outside the visual modality. Our paradigm demonstrates that neural signatures of evidence accumulation like CPP build-up rate may reflect only task performance capacity itself and not metacognitive measures of confidence. Our results thus provide a strong contribution to the literature that may be followed up in future experiments, contributing to a more holistic understanding of perceptual metacognition.

## Supporting information

S1 Fig Relationship between task performance (d’) and metacognitive sensitivity (meta-d’). Participants are categorized according to musical aptitude (group 1 = no absolute pitch and no musical training; group 2 = no absolute pitch, some musical training; group 3 = absolute pitch and musical training).
(TIF)

S2 Fig ERPs as a function of tonal distance bin at electrode Pz alone. **(A)** Stimulus-locked ERP. **(B)** Response-locked ERP.
(TIF)

S3 Fig Topoplot of response-locked ERP from 0 to 500 ms. (TIF)

S4 Fig Relationship between CPP build-up rate and musical sophistication score across subjects, split as a function of normalized reaction time (slow, medium, fast) within each subject. (TIF)

S1 File Participant self-questionnaire. (DOCX)
