# Supplementary figures and images for: Behavioral and neural measures of confidence using a novel auditory pitch identification task
Source: PLoS One. 2024 Jul 1;19(7):e0299784. doi: 10.1371/journal.pone.0299784 (PMC11216601; doi:10.1371/journal.pone.0299784)

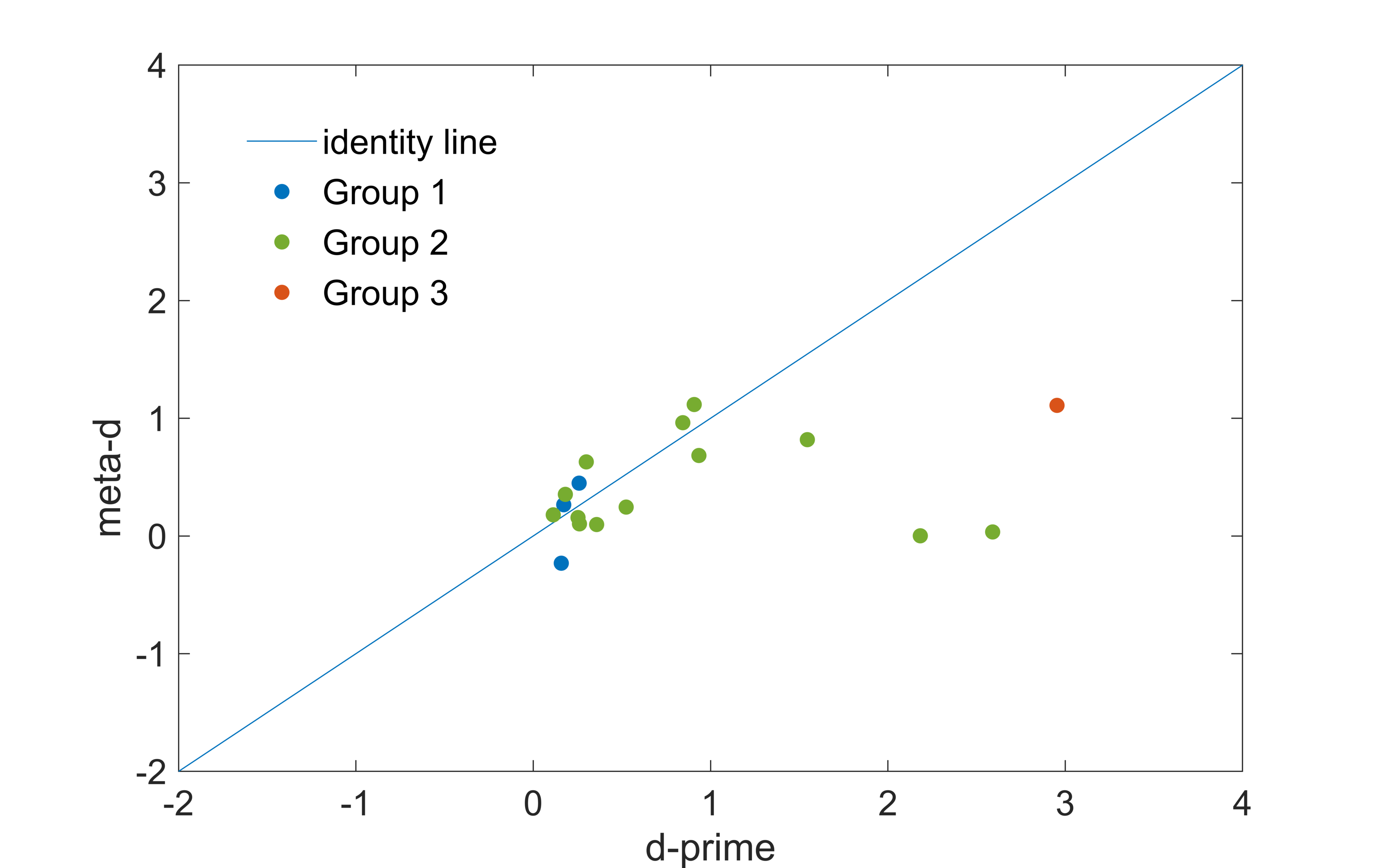

Supplement: S1 Fig — Participants are categorized according to musical aptitude (group 1 = no absolute pitch and no musical training; group 2 = no absolute pitch, some musical training; group 3 = absolute pitch and musical training). (TIF) [file pone.0299784.s001.tif]

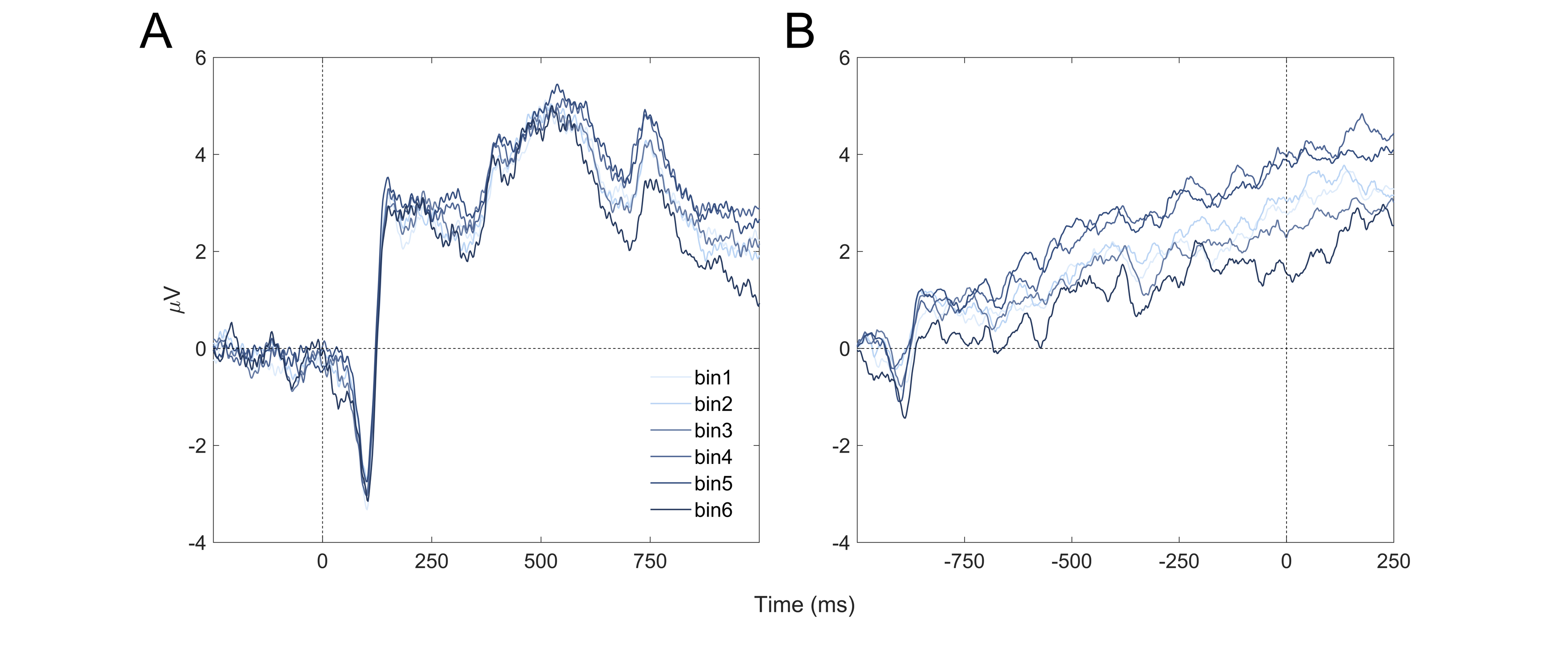

Supplement: S2 Fig — (A) Stimulus-locked ERP. (B) Response-locked ERP. (TIF) [file pone.0299784.s002.tif]

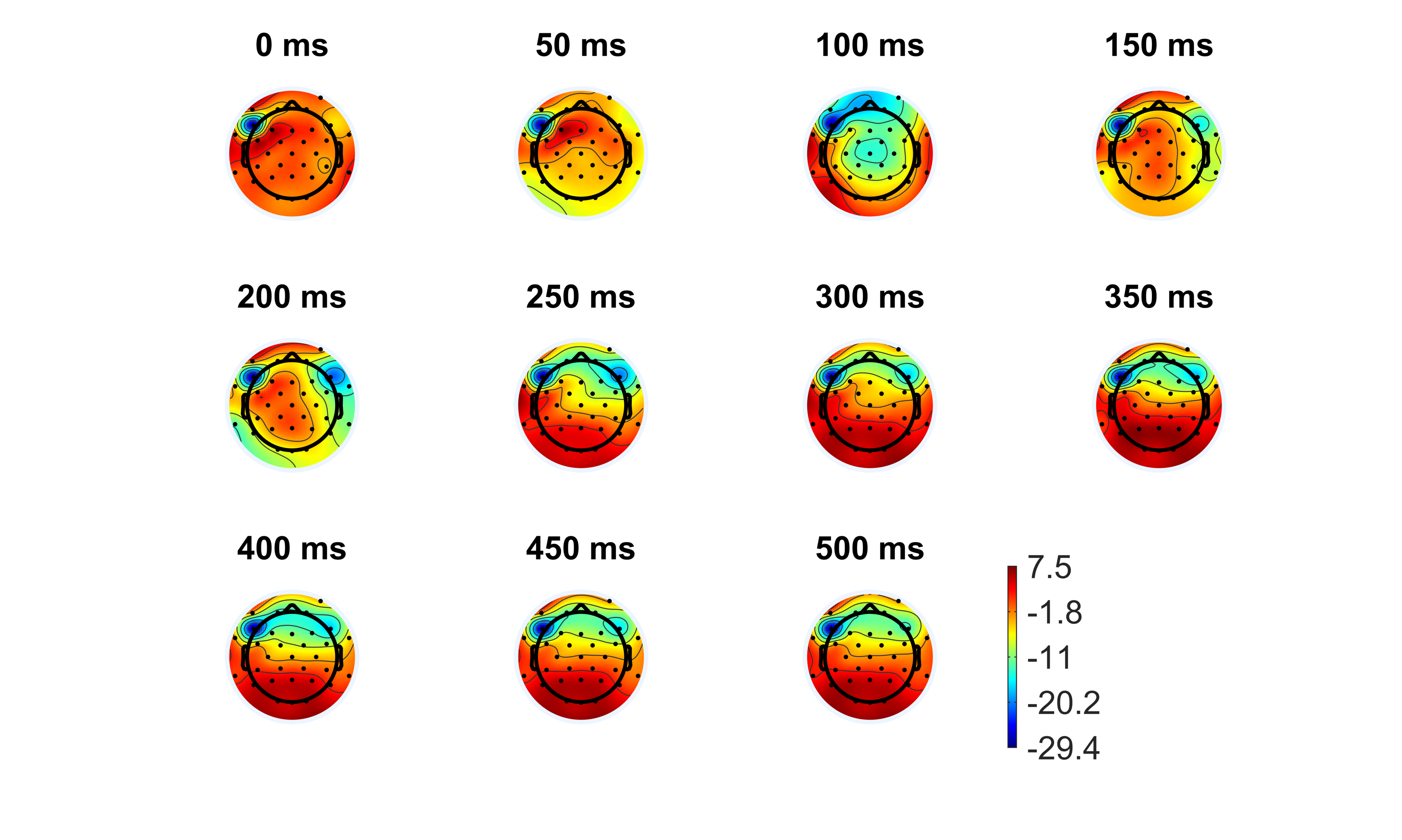

Supplement: S3 Fig — (TIF) [file pone.0299784.s003.tif]

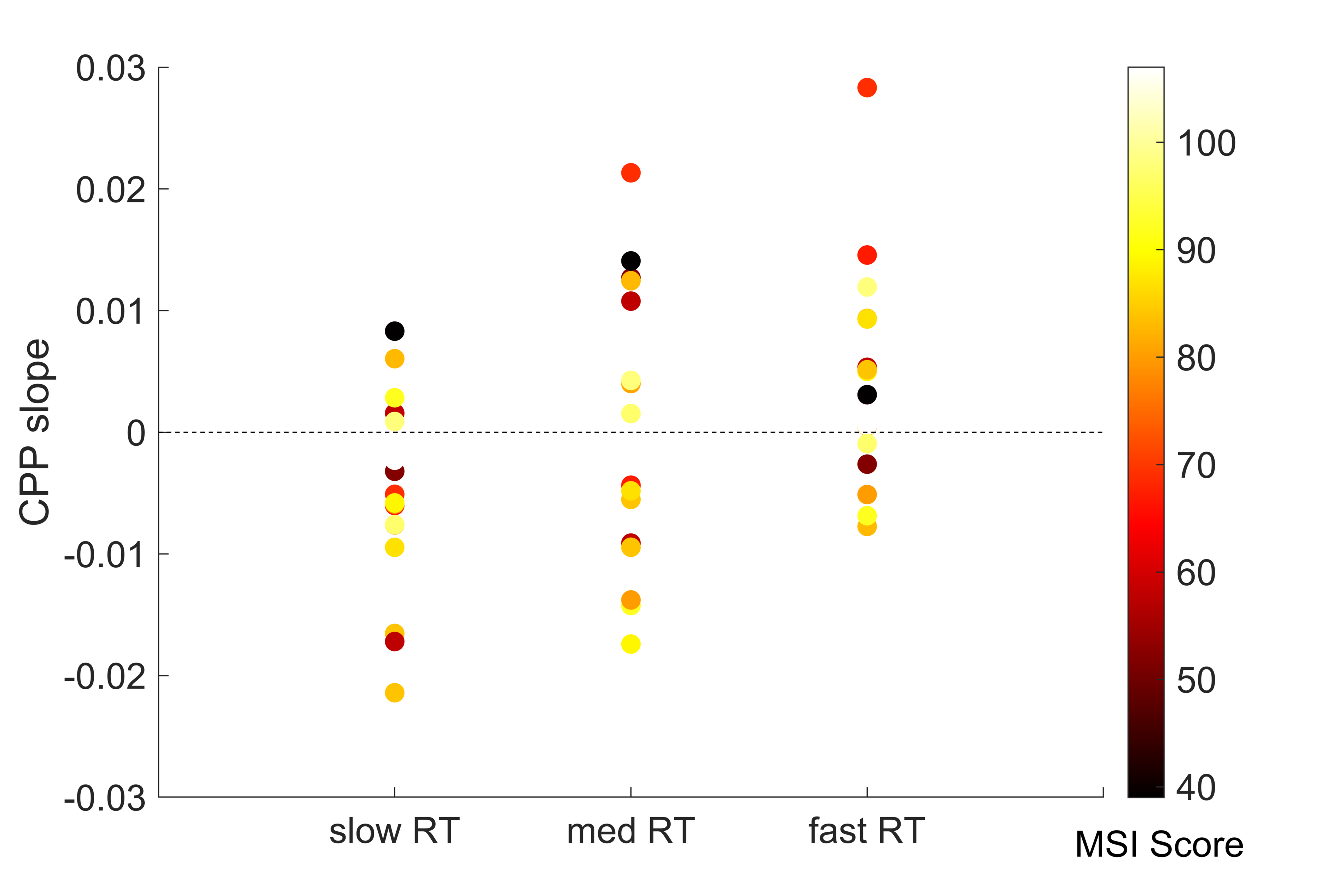

Supplement: S4 Fig — (TIF) [file pone.0299784.s004.tif]
