# Supplementary material for: Behavioral and neural measures of confidence using a novel auditory pitch identification task
Source: PLoS One. 2024 Jul 1;19(7):e0299784. doi: 10.1371/journal.pone.0299784 (PMC11216601; doi:10.1371/journal.pone.0299784)
Supplement: S1 File — (DOCX) [file pone.0299784.s005.docx]

**Auditory Pitch Discrimination Task:**

**Subject Self-Questionnaire**

**Name:** ____________________________ **Gender:** ________ **Age:** ______

**# of Languages Spoken:** ____

**List the number of languages spoken (if not applicable, write N/A):**

_____________________________________________________________

_____________________________________________________________

**Musical training? (Y/N)**

**How many years of formal education have you had? (if not applicable, write N/A)**

_____________________________________________________________

**At what age did you begin formal education? (if not applicable, write N/A)**

_____________________________________________________________

**Amount of training? (average hours of practice per week) (if not applicable, write N/A)**

_____________________________________________________________

**Primary Instrument:**

_____________________________________________________________
